# The adherence/resource priming paradigm – a randomised clinical trial conducting a bonafide psychotherapy protocol for generalised anxiety disorder

**DOI:** 10.1186/1471-244X-14-49

**Published:** 2014-02-20

**Authors:** Christoph Flückiger

**Affiliations:** 1 Department of Psychology, University of Zürich, Binzmühlestr. 14/18, CH-8050 Zürich, Switzerland

**Keywords:** Generalised anxiety disorder, Cognitive behaviour therapy, Adherence

## Abstract

**Background:**

Bonafide psychotherapy is an effective treatment for generalised anxiety disorder compared to no-treatment. Rather than creating increasing numbers of new overall treatment-packets within a medical meta-model, an additional approach to investigating clinical research designs may be to increase the understanding of already effective psychotherapies. Treatment manuals and protocols allow a relatively high degree of freedom for the way therapists implement the overall treatment manuals. There is a systematic lack of knowledge on how therapists should customise these overall protocols. The present study experimentally examines three ways of conducting a bonafide psychotherapy based on a 15 session time-limited cognitive-behavioural therapy (CBT) protocol and their relation to the therapists’ protocol adherence and treatment efficacy.

**Methods/design:**

This trial will investigate three different methods of customising a bonafide CBT-protocol using dyadic peer-tutoring methodology (primings). The individuals with GAD will be randomly assigned to one of three priming conditions (resource priming, supportive resource priming, or adherence priming). The participant treatment allocation will be performed randomly. Therapists will be assigned to a peer-tutoring partner and priming condition based on a mutual agreement. Treatment outcomes will be assessed at the following times: observer based in-session outcomes, session-by-session post-session outcomes, treatment outcome at post assessment and treatment outcome at 6-month follow-up.

**Discussion:**

The proposed trial addresses the clinically relevant question of how to customise a bonafide psychotherapy protocol using tandem peer-tutoring methodology (three priming conditions). Through the development and testing of the proposed priming procedures, this study describes levels of adherence and how to conduct an overall treatment protocol in a more systematised way.

**Trial registration:**

From ClinicalTrials.gov Identifier: NCT02039193.

## Background

In European countries, the lifetime prevalence of generalised anxiety disorder (GAD) varies from 5 to 10% [1-5]. Uncontrollable worrying as a primary symptom of GAD constitutes a maladaptive cognitive strategy to avoid experiencing anxiety [6,7] and emotional states in general [8,9]. Individuals with GAD show deficits in detecting and regulating emotional states, which may accelerate a positive feedback circuit between general stress symptoms and pathological worrying [10-12]. Finally, experiential avoidance may lead to a restriction in proactive behaviours as individuals become focused on avoiding events and situations rather than pursuing activities that are consistent with their personal values [13,14], which may be related to the hypersensitivity of the Behavioural Inhibition System seen in individuals with GAD [15].

Bonafide psychotherapy is an effective treatment for GAD compared to no-treatment and treatment as usual [16-18], but also more generally for individuals that suffers from anxiety and depression [19]. For cognitive-behavioural therapy (CBT), there are a number of treatment manuals/protocols, e.g. psycho education, relaxation techniques, cognitive restructuring of (meta-) cognitions, (imagery-) exposure, and in vivo confrontation, that primarily reference standard techniques to correct and compensate GAD symptoms [20-24]. In addition, strengths-oriented strategies aim to capitalise on the patient’s pre-existing resources, such as the patient’s individual and interpersonal abilities and readiness. Such strategies, e.g. acceptance strategies [25], motivational interviewing [26], and well-being therapy [27] and, more generally, solution-focused interventions [28], positive interventions [29], resilience-focused interventions [30] and resource-oriented approaches [31-34], have recently been discussed for GAD treatment. Such strengths-oriented tools, however, may be based on more general counselling and consulting psychology principles, such as making hope explicit [35,36] or understanding the client’s functional behaviours [37-39].

The separation into compensation- and capitalisation-oriented strategies probably a false dichotomy, and psychotherapy (socratic) dialogues often simultaneously respond to the participants’ targeted weaknesses and strengths in general [40-43] but also to GAD more specifically [44]. Psychotherapy dialogues can be seen as a highly interactive and responsive treatment through which therapists and patients work together to achieve well-specified treatment goals that take into consideration the patients’ entire living environment, including strengths and weaknesses [45-47].

Rather than creating increasing numbers of new overall treatment-packets within a medical meta-model, an additional approach to investigating clinical research designs may be to increase the understanding of already effective psychotherapies [45,48-50]. Moreover, psychotherapy research is moving from legitimation- and competition-oriented investigations to more comprehension-oriented process-outcome based research [51]. Treatment manuals and protocols allow a relatively high degree of freedom for the way therapists implement the overall treatment manuals. There is a systematic lack of knowledge on how therapists should customise these overall protocols [31,52,53]. The present study experimentally examined three ways of conducting a cognitive-behavioural therapy (CBT) protocol and their relation to the therapists’ protocol adherence and treatment efficacy.

### Aims of the trial

This trial will investigate three different methods of customising bonafide psychotherapy based on a well-introduced CBT-protocol by Zinbarg, Craske and Barlow [24] using dyadic peer-tutoring methodology (primings). The participants will be randomly assigned to three priming conditions: (a) adherence priming; (b) resource priming or (c) supportive resource priming [32,39].

The main research questions are as follows:

(1) In-session outcomes. Using videotapes, observer-based video analyses will be conducted: (a) Do the resource priming conditions show comparable observer-based adherence of the therapist in comparison to the adherence priming condition [54]? (b) Do the resource priming conditions result in more resource-activating micro-interventions than the adherence priming condition [55]? (c) Are the in-session processes predictors and mediators of session and therapy outcomes [43]?

(2) Post-session outcomes. Are there differences in the post-session outcomes for the 3 different priming conditions? Furthermore, are the post-session outcomes also symptom change predictors and mediators of therapy outcome [56]?

(3) Treatment outcomes. Do the resource priming conditions have comparable efficacy on GAD outcomes, general outcomes and dropout rates in comparison to the adherence priming condition?

## Methods/design

**Figure 1:**
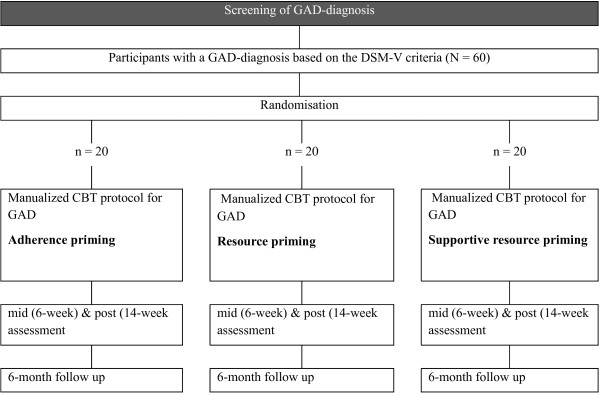
Flow chart of the study design.

This study is a randomised controlled trial with three active treatment arms. This trial will be conducted at the Swiss psychotherapy outpatient clinic in the Department of Psychology at the University of Zürich. Figure 1 depicts the 3 × 4 design with one between-subject factor (resource priming, supportive resource priming, or problem priming) and one within-subject factor (time: pre, mid, post, or follow-up).

### Participants

#### Inclusion/exclusion criteria

Participants will be included in the study if they: (a) are 18 years of age or older; (b) agree to the informed consent, (c) have sufficient knowledge of German; and (d) and fulfil the diagnostic criteria of GAD DSM-IV. Participants will be excluded for the following reasons: (a) they have a score of 2 or higher on the suicide item of the BDI and/or are found to have active suicidal plans during the diagnostic screening interview, (b) they are currently taking a psychotic or bipolar disorder medication, or (c) they are currently receiving treatment from a professional psychotherapist. Prescribed medications for anxiety or depressive disorders do not lead to exclusion from the study, if the dosage has remained constant for at least one month. The presence of comorbidities does not result in exclusion from the study, if GAD is in the foreground according to the severity rating of the Diagnostic Interview for DSM-diagnoses.

#### Recruitment

Participants will be recruited by means of advertisements in newspapers and through internet forums. High-circulation newspapers are delivered for free through the Swiss public transport system. Individuals interested in participating in the study will contact the study office via SMS, e-mail or phone. Positively screened patients will be invited for an intake assessment to determine whether they will be included or excluded using a standardised interview. Participants that are not screened positively will be informed of more appropriate treatments via a phone call or, if requested, a face-to-face contact.

#### Randomisation and treatment allocation

After meeting the inclusion criteria, patients will be randomly assigned to one of the three conditions (adherence priming, resource priming, or supportive resource priming). Treatment allocation is performed using an online application for full randomisation. In this way, we aim to ensure that the trial arms are balanced with respect to the patients’ baseline characteristics. Randomisation will be conducted by two independent research assistants. Because all patients will be treated using the same CBT-manual, patients are blinded to their treatment allocation and are not informed about the randomisation procedure.

### GAD treatment protocol

*CBT-manual* by Zinbarg, Craske and Barlow (MAW-packet) [24]: CBT for GAD typically consists of psycho-education of generalised anxiety disorder, relaxation training (RT), cognitive restructuring (CR) and some in-vivo situational exposure for patients with overt behavioural avoidance [20,22]. Furthermore, imagery exposure as a GAD-specific form of in-sensu exposition will be applied to reduce experiential avoidance. The manualized therapy follows a usual treatment format of 14 50-minute sessions and a booster session after 6 months (15 sessions in total).

### Tandem peer-tutoring (priming)

To investigate various methods of conducting a standardised CBT-protocol, all therapists are tutored in peer dyads (tandem peer-tutoring). Immediately before sessions 1 to 5, the therapists are required to contact the tandem-partner face-to-face or on the phone to discuss the upcoming session in a 5 to 10 minute brief conversation (primings; for a comparable procedure see Flückiger and Grosse Holtforth [32]). There are three tandem peer-tutoring conditions:

(1) *Adherence priming*: Immediately before sessions 1 to 5, a therapist conducts five-minute conversation about how to implement the disorder-specific interventions that are described in the treatment protocol. These communications are focused on the therapist’s understanding of the patients GAD and any related comorbidities and how those issues can be addressed in the prescriptive treatment protocol.

(2) *Resource priming*: Immediately before sessions 1 to 5, a therapist conducts a five-minute conversation with the participant about how to implement strengths-based micro-interventions in the upcoming session. Strengths-based micro-interventions allow therapists to focus on a patient’s pre-existing strengths and abilities, subtle changes and improvements to those strengths and abilities during therapy (potential resources), and motivational preparedness, readiness and goals (motivational resources) [39,57].

(3) *Supportive resource priming*: The supportive resource priming condition uses the same protocol as the resource priming condition (5 brief tandem peer-tutorings). The only difference in the procedure is that the therapists are allowed to integrate a helpful significant person from the patients entourage (such as a partner or best friend) during sessions 1 and 7 to encourage them to support the patient in adhering to their treatment plan (active integration of interpersonal resources). However, the integration of a significant person does not affect the CBT-treatment protocol.

### Therapists

Twelve advanced trainees with at least 2 years post-graduate training will be recruited from local psychotherapy-training centres. The majority of those therapists have experience as study therapists in a prior randomised controlled trial (ClinicalTrials.gov Identifier: NCT01012856). All of the therapists will participate in an initial 16-hour workshop presented by the (co-)developer of the treatment manual (Zinbarg) [24]. In addition to the peer-tutoring (primings), the therapists will be regularly supervised in small groups on a 14-day basis. The supervision is conducted in mixed groups for all three priming conditions. All of the supervisors also participate in the initial 16-hour workshop. To respect and coordinate the therapists’ preferences (e.g., preferences in working days and time schedules) they will be assigned in a joint face-to-face session at the beginning of the study. All of the therapists will give verbal and written consent for their selected peer-tutoring partner and priming condition. To control for potential effects of particular therapists, we will investigate the therapists’ self-reported interpersonal strengths and problems, their attachment quality and their therapeutic attitudes along with their allegiance to the underlying CBT-protocol.

### Assessments

For an overview of the assessments see Table 1. At intake, GAD-diagnosis and its core symptomatology will be identified according to the structured interview section for GAD (DIPS; [58]). Furthermore, GAD-criteria are assessed using self-reports. The individual worries are identified using the Penn State Worry Questionnaire (PSWQ; [59,60]) and the Worry Domain Questionnaire (WDQ; [60,61]). Mental disorders on Axis I and II are assessed using face-to-face diagnostic interviews (Strukturiertes Klinisches Interview für DSM, SKID-I/II; [62]).

**Table 1:** Schedule of measures

**Measures**	**Measurement waves**					
	**Ass.**	**Pre**	**Sess-by-Sess**	**Mid**	**Post**	**FU**
*Eligibility*						
Structured Interview for DSM (SCID) [62]	+					
GAD-section of the (F-DIPS) [58]	+					
GAD-criteria self-report [62]	+					
WDQ [60,61]	+					
*GAD-outcomes*						
Beck Anxiety Inventory (BAI) [63,64]		+	1-15	+	+	+
Penn State Worry Questionnaire (PSWQ) [59,60]	+	+		+	+	+
State –Trait Anxiety (STAI) [65,66]		+		+	+	+
*General-outcomes*						
Premature termination			1-15			
Beck Depression Inventory II (BDI-II) [67,68]	+	+		+	+	+
Brief Symptom Inventory (BSI) [69,70]		+		+	+	+
BIS/BAS scales [71,72]		+		+	+	+
Inventory of Interpersonal Problems (IIP-64) [73,74]		+		+	+	+
Inventory of Interpersonal Strengths (IIS-64) [75,76]		+		+	+	+
Resource potential questionnaire (RES) [75,77]		+		+	+	+
*Self-report process-measures*						
Working Alliance Inventory - Patient (WAI-P) [78,79]			1-15			
Working Alliance Inventory – Therapist (WAI-T) [78,79]			1-15			
Bern Post-Session Report – Patient (BPSR-P) [80]			1-15			
Bern Post-Session Report – Therapist (BPSR-T) [80]			1-15			
*Video observer ratings*						
Adherence rating			2,5,8,11			
Resource-oriented Microprocess Analysis – Patient (ROMA-P) [55]			2,5,8,11			
Resource-oriented Microprocess Analysis – Therapist (ROMA-T) [55]			2,5,8,11			
Level of explication [81]			2,5,8,11			
*Therapists’ measures*						
Inventory of Interpersonal Problems (IIP-64) [73,74]	+_1_					
Inventory of Interpersonal Strengths (IIS-64) [75,76]	+_1_					
Measure of Attachment Quality (MAQ) [82,83]	+_1_					
Therapists’ allegiance self-report	+_1_					
Therapeutic Attitude Scale (TASC), therapeutic style section [84]					+_2_	

_1_At the beginning of the study, _2_At the overall end of the treatment.

The PSWQ, Beck Anxiety Inventory (BAI; [63,64]) and the State-Trait Anxiety (STAI; [65,66]) questionnaire will used to measure GAD-outcomes. Premature termination from the trial, interpersonal problems and strengths inventories, general symptom severity, the behavioural inhibition and activation scale, as well as the resource potential questionnaire will be used to measure general outcomes (see Table 1). The outcome measures are taken at intake, directly after session 6 (end of primings), session 14 (post-assessment), and at the 6-month follow-up.

The following process measures are examined: (a) Post-session reports: alliance [78,79], post-session outcomes [80] and symptom change are conducted based on a session-by-session assessment from session 1 to 15 and (b) In-session processes: using the recorded videotapes, adherence ratings for the CBT-program [54], the Resource-Oriented Microprocess Analysis (ROMA-P/T; [55]) and the level of explication scale by Sachse [81] are conducted at sessions 2, 5 and 8.

### Study from the participants view

**Figure 2:**
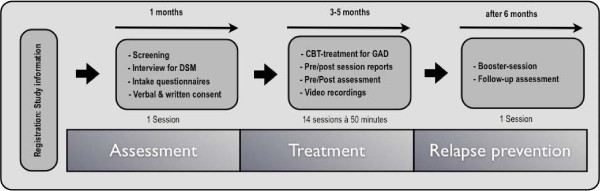
Study from the participant’s point of view.

After contacting the study administration, the information given to the participants will contain a precise description of the inclusion and exclusion criteria, the structure of the treatment manual as well as the data collection procedure, including the procedure for the videorecordings (Figure 2). Confidentiality of the collected data will be confirmed. Voluntariness of participation will be emphasised by explaining the opportunity to terminate the treatment at every timepoint during treatment and the opportunity to cancel the use of any data collected. Furthermore, an insurance policy for possible negative outcomes resulting from participation in clinical trials will be contracted.

### Statistical analysis

To handle the hierarchical data structures (sessions at level 1 nested within patients at level 2 and therapists at, level 3), a hierarchical linear model (HLM) with time as a within-groups factor and the treatment condition as a between-groups factor will be used for the main research questions [85]. The analyses will be conducted on the intention-to-treat sample as well as on the completer sample. To investigate if patients and therapists have effects on the therapy outcomes, the patients’ and therapists’ pre-treatment characteristics will be investigated as outcome predictors at levels 2 and 3.

### Sample size

Based on a power analysis with G*Power [86], the optimal sample size with an Alpha-error of 5%, a Beta-error of 80% and a correlation coefficient for the repeated assessments of of r = .30 is 60 participants, i.e., 20 participants in each priming condition.

### Ethical review

This study protocol is approved by the Ethical Committee of Canton Zurich (KEK 2011–0475) and the Ethical Committee of the Philosophical Faculty of University of Zurich (PhiF-EK_20.1.2012).

## Discussion

Bonafide psychotherapy is an effective treatment for individuals who suffer from GAD and its comorbidities in comparison to no-treatment and treatment as usual [16,17,18]. Rather than creating increasing numbers of new overall treatment-packets within a medical meta-model, an additional approach may be to increase the understanding of already effective psychotherapies [45,48-50]. The psychotherapy field is moving from legitimation and competition focused research to a more comprehension-oriented process-outcome research methodology [47,51]. Treatment manuals and protocols allow a relatively high degree of freedom for the way therapists implement the treatment protocols. The present design experimentally examines the therapists’ within-protocol variability and its relation to treatment outcome. More specifically, three methods of conducting the same treatment protocol are compared to each other using tandem peer-tutoring methodology (resource priming, supportive resource priming, and adherence priming).

The hierarchical structure of the design allows the simultaneous examination of the patients’ contributions and the therapists’ contributions. In contrast to double blinded medication trials, therapists’ and patients’ are informed and actively involved in the psychotherapeutic treatment plans. This involvement is not a bias that has to be eliminated and it might be well an active ingredient of a successful psychotherapy that the therapists’ and patients’ take a proactive, responsive role in the treatment [37,87]. The present design allows for the experimental investigation of some potentially meaningful aspects of this proactive variability.

### Bias minimization

#### Participants

Patients will be randomly assigned to conditions in order to reduce potential biases of participant characteristics. The inclusion and exclusion criteria allow for a relatively homogeneous group of individuals; the primary GAD diagnoses, comorbidities with substance use disorders and severity of depression along with age, sex and gender will be tested as potential confounders.

In traditional psychotherapy trials, where two or more treatments protocols are compared to each other, the patients’ are informed about the various randomised treatment conditions of the active treatments (e.g., psychotherapy vs. waiting list). In the present trial, the patients’ are treated with the same treatment protocol, and, thus the randomisation procedure essentially does not affect the overall treatment plan. Referring to the pre-existing standards, the patients can be blinded to the randomised therapist conditions in order to reduce potential biases from the outcome expectations of the patients.

#### Therapists

A potential bias due to allegiances of the therapists is a concern, especially in human treatments where inductions of outcome expectations can be problematic. To minimise this potential bias, therapists will be allocated to the peer-tutoring partners based on common consent. Furthermore, they will be supervised in mixed groups, where the priming condition plays no explicit role and the supervisors are advised to focus on therapist skills independent of the underlying priming conditions. Nonetheless, because the therapists are informed about the treatment protocol, the allegiances of the therapists to specific CBT protocols will be declared and considered in the statistical analyses.

Keeping the above mentioned strengths and limitations of the present design in mind, the proposed trial addresses the clinically relevant question of how to customise a bonafide psychotherapy using tandem peer-tutoring methodology (three priming conditions). Through the development and testing of the proposed priming procedures, the study could potentially define levels of adherence for conducting an overall treatment protocol more appropriately [52,88].

## Competing interests

The author declares that he has no competing interests.

## Authors’ contributions

CF is the principal investigator running the study primary over a period of 3 years.

## Pre-publication history

The pre-publication history for this paper can be accessed here:

http://www.biomedcentral.com/1471-244X/14/49/prepub
